# Erector Spinae Plane Block in Open Appendectomy: A Case Series

**DOI:** 10.1155/cria/3705137

**Published:** 2025-06-25

**Authors:** Jassim Rauf, Mohammad Mohsin A. M. Haji

**Affiliations:** ^1^Department of Anesthesiology, ICU & Perioperative Medicine, Hamad General Hospital, Hamad Medical Corporation, Doha, Qatar; ^2^College of Medicine, Qatar University, Doha, Qatar

**Keywords:** acute appendicitis, fascial plane block, levobupivacaine, morphine, regional anesthesia

## Abstract

**Background:** Open appendicectomy is a painful procedure requiring multimodal analgesia. Various fascial planes have been used successfully in the past as part of this multimodal analgesia like TAP block, quadratus lumborum block, and even local infiltration. All of these analgesic regimes have been shown to reduce intra- and postoperative opioid consumption. In this case series, we elaborate on the effects of ESPB on the consumption of opioids in the intra- and postoperative periods of two patients undergoing open appendicectomy.

**Case Series:** Two American Society of Anesthesiologists (ASA) Emergency (1E) patients scheduled to undergo open appendicectomy were consented to receive ESPB after receiving GA. The block was performed at the transverse process at T12 level, on the right side with 30 mLs of 0.25% levobupivacaine. One patient received intra-op IV paracetamol and ketorolac, while the other patient did not receive paracetamol as he already received it in the ward and ketorolac was withheld due to high creatinine. The surgeries went uneventful. Both patients reported 0 NRS pain score in PACU. One of the patients recorded 0 NRS pain score till discharge, and the other reported an NRS score of 5/10 at 19 h 30 min. Both were discharged from the hospital after 24 h.

**Conclusion:** ESPB has a potential opioid sparing effect and can provide a total morphine free analgesia both in the intra- and postoperative period in the setting of an open appendicectomy.

## 1. Introduction

Open appendicectomy is a painful procedure. Though with the advent of Laparoscopy, the number of open surgeries has gone down but still at times patients do require an open approach due to surgical, anesthetic or patient factors. Regional anesthesia, with the introduction of newer blocks, has become part of multimodal analgesia in many surgeries. It is an established fact that addition of regional anesthesia does reduce intra- and postoperative opioid consumption in many surgeries [1]. Erector spinae plane block (ESPB) was first described by Forero et al. [2] for patients with neuropathic thoracic pain. The block, as proved in many studies, has become popular as it not only provides somatic but also visceral analgesia by spreading to its surrounding structures, predominantly the dorsal horn, inconsistent spread to ventral horn and the paravertebral spread and occasionally to the epidural space [3]. There is evidence that ESPB has a role in open abdominal surgeries. Mohamed et al. [4] reported in a randomized controlled Trial (RCT) that ESPB reduced intra- and postoperative fentanyl requirements after open abdominal hysterectomy. Abu Elyazed et al. [5] in a RCT proved that ESPB significantly reduced the postoperative pain scores and intra- and postoperative opioid consumption in open epigastric hernia repair.

In this case series, we will look at the outcomes of two patients who received ESPB for open appendectomy. The surgeons decided to go for open appendicectomy as preoperative abdominal US confirmed an appendiceal mass which could not have been retracted laparoscopically.

Both patients were consented for GA and ESPB.

## 2. Case Series

### 2.1. Case 1

38-year-old male, ASA 1E with no past medical and surgical history diagnosed with acute appendicitis was scheduled to undergo open appendectomy. The patient weighed 80 kg with a Body Mass Index (BMI) of 28 kg/m^2^. The patient had received 1 g of paracetamol intravenous (IV) and reported a maximum pain on numerical rating scale (NRS) of 5/10 upon arrival in the preholding area of the operating theater (OT).

Intraoperatively, after connecting to standard ASA monitors, GA was induced using IV propofol 2.5 mg/kg, fentanyl 100 mcg, and rocuronium 0.5 mg/kg. Orotracheal intubation was performed with an Endotracheal Tube (ETT) sized 7.5.

After induction the patient was positioned in the left lateral position and an ultrasound-guided (US) ESPB was performed on the right side, at T12 level of the spinal cord.

Anesthesia was maintained on sevoflurane with a minimum alveolar concentration (MAC) of 1.2% throughout the case. Patient received IV dexamethasone 8 mg at induction and then IV paracetamol 1 g, ketorolac 30 mg during the surgery. At the end of an uneventful appendectomy, the patient received 4 mg of ondansetron and 200 mg of sugammadex. The patient was extubated and transferred to Postoperative Anesthesia Care Unit (PACU).

In PACU patient was comfortable, hemodynamically stable and reported a pain score of 0/10 on NRS. Patient's vital signs are presented in Table 1, both intraoperatively and during PACU stay. In the postoperative period, the patient did not complain of any pain till 19 h 30 min postblock performance and reported a NRS pain score of 5/10. Patient received IV paracetamol at that time point. There after the patient was discharged after 24 h.

### 2.2. Case 2

33-year-old male, ASA IE with no past medical and surgical history, presented with acute abdomen and was diagnosed with acute appendicitis. Patient was scheduled for open appendectomy. The patient weighed 83 kg with a BMI of 29 kg/m^2^. Preoperatively, the patient was prescribed for regular paracetamol, hence before arrival into the operating room, had received two doses of 1 g IV paracetamol at an interval of 6 h, of which 1 dose was given just 2 h before arrival into the OT area. Preoperatively on the ward, maximum NRS pain reported by the patient was 5/10.

In the OT, the patient was connected to standard ASA monitors. Patient was preoxygenated and induction was done with fentanyl 100 mcg, Propofol 2.5 mg/kg and rocuronium (0.6 mg/kg). Orotracheal intubation was performed and patient thereafter was placed in the left lateral position for block performance. Like the previous patient, this patient also received an US guided ESPB block at T12 level on the right side.

Anesthesia was maintained with sevoflurane with a MAC of 1.5. Patient did not receive any paracetamol as he was not due. We did not administer any ketorolac as patient had high serum creatinine level 117 umol/L (normal range 62–106 umol/L). At the end of the surgery, patient received IV ondansetron 4 mg and sugammadex 200 mg, was extubated uneventfully and was transferred to PACU.

Postoperatively in the PACU, the patient reported a NRS pain score of 0/10 in PACU and had a very stable hemodynamic profile. The intraoperative and PACU hemodynamic profile of the patient at different time points is presented in Table 2. In the next 24 h, patient received 4 more doses of 1 g IV paracetamol at 6 h interval, as he was prescribed regular paracetamol. Patient recorded no pain during this period; his creatinine levels normalized postsurgery and were discharged after 24 h of surgery without any further sequel.

## 3. Block Performance

After intubation, patient was positioned in the left lateral position. Under full aseptic conditions, the spinous process (SP) of the C 7 vertebra was identified using both US and landmark techniques. Palpation of C7 SP and downwards till T7 SP was felt. Then a line was drawn adjoining the lower edge of both scapulae. By combining these 2 methods, we wanted to eliminate any doubt about the anatomical level. Thereafter a 6–13 mHz linear probe (Sonosite M-Turbo ultrasound device, Fujifilm, Sonosite, WA, USA), was placed on the T7 SP in a parasagittal direction and was moved caudally till T12 SP was identified. Next, the probe was moved laterally to the right side and the transverse process (TP) of the T12 was identified. At this point an 80 mm 22 g Sonoplex STIM needle was inserted in a craniocaudal direction, in plane with the US probe, till the tip of the needle came in contact with the TP of T12. One mL of normal saline was injected to confirm that the tip was in the erector spinae muscle plane. Once the rise of the muscle was visualized, 30 mLs of 0.25% levobupivacaine was injected with intermittent aspiration.

## 4. Discussion

While there is evidence that ESPB has opioid sparing effects in open surgeries some trials report that its role is limited to only a few hours in the postoperative period. Jeong et al. [6] in a RCT showed that an ESPB for open gastrectomy was effective only for the first 3 hours in the postoperative period. Dost et al. [7] did a RCT on ESPB in patients undergoing open radical prostatectomy and reported that the block was effective only in the first hour of the postoperative period. There have also been some comparative studies, comparing quadratus lumborum (QL) block with the ESPB [8] in patients undergoing open nephrectomy. The authors concluded that both blocks are equally effective in open nephrectomies and should be done according to the level of expertise of the anesthesiologist.

The most widely used block for open appendicectomy is transversus abdominis plane (TAP) block [9, 10] and has demonstrated a significant reduction in the perioperative pain scores and opioid consumption. QL block has been shown to provide not only somatic analgesia but also visceral analgesia when used for lower abdominal surgeries. Mullins et al. [11] reported in their case report, where they used EMG for a bilateral US guided QL block for an open appendectomy, the patient remained pain free for 18 h in the postoperative period with blockade of sensory nerves T6 to L2. However, QL block higher levels of expertise as it is a relatively deep block.

The appendix derives its nerve supply from T10 level [12]. ESPB is part of the PLAN A blocks as per The Royal college of Anesthetists for its safety profile. It is a very superficial block and easy to perform. There is very strong evidence now that ESPB not only covers somatic pain but also provides visceral pain relief. Adhikary et al. demonstrated in their cadaveric study that ESPB with 20 mLs of dye spread through neuroforamina and into the epidural space as well [13]. The authors also concluded that levels stained varied from 2 to 5 and that ESPB showed extensive vertical spread as well when compared to the retrolaminar block. A case report published in July 2022, showed that ESPB can be an effective mode of analgesia for acute appendicitis pain relief in the ED setting [14] injecting LA at the level of L1. The case report demonstrated that the block at L1 was enough to spread to T10 level in order to provide visceral pain relief. The surgical incision for open appendectomy involves the L1 and L2 dermatomes and at times even T12. De Cassai and Tonetti reported that volume required to cover a single dermatome can range between 2.5 and 6.6 mLs with a median value of 3.4 mLs per dermatome [15]. Based on the finds of Adhikary et al. and De Cassai and Tonetti our decision to inject 30 mLs at T12 level was right as it provided adequate somatic and visceral pain relief. The spread of LA depends on the point of injection, LA volume and the length of the TP [16]. However, the spread can be highly variable as described by Tulgar et Al in their study [17].

Our first patient remained pain free for up to 19 h 30 min. When compared to Mullins et al.'s case report where the patient received bilateral QL block, intraoperative morphine and also one dose of tapentadol 50 mg at the 8 h postoperative time point, both of our patients received unilateral ESPB and did not receive any morphine intraoperatively and/or any opioid in the postoperative period till discharge.

ESPB is possibly the most investigated fascial plane block. More and more evidence is now coming out regarding the use of additives in LA that provide prolonged analgesia with ESPB that is fentanyl, dexamethasone, magnesium etc. More recently multiple level versus single level ESPB has shown more higher quality of analgesia, but some studies show that it is beneficial only in the early postoperative hours [18]. In an open appendicectomy since there is a single vertical incision hence the authors did not deem it necessary for a multiple point ESPB.

Further retrospective studies or randomised controlled trials are needed to support our findings.

## 5. Conclusion

Our case series is the first to demonstrate that a single shot ESPB at T12 level for open appendectomy can provide totally morphine free analgesia in the intra- and postoperative period.

## Figures and Tables

**Table 1:** Case 1 perioperative vitals.

Time point	HR	BP	SpO_2_
5 min pre-GA induction	70	121/69 (MAP 85)	100
5 min post-GA induction	98	99/70 (MAP 79)	100
5 min preblock	99	120/61 (MAP 99)	100
5 min postblock	90	119/81 (MAP 91)	100
5 min postincision	79	120/70 (MAP 85	100
PACU time 00 min	82	127/82 (MAP 97)	100
PACU time 15 min	80	130/78 (MAP 95)	100
PACU time 30 min	81	128/70 (MAP 89)	99
PACU time 45 min	80	130/72 (MAP 91)	99

*Note:* HR: heart rate beats per minute; BP: blood pressure in mmHg systolic/diastolic; MAP: mean arterial pressure in mmHg; SpO_2_: oxygen saturation in percentage. PACU readings are recorded at 15 min interval from the time of arrival into PACU.

Abbreviation: PACU, Postoperative Anesthesia Care Unit.

**Table 2:** Case 2 perioperative vitals.

Time point	HR	BP	SpO_2_
5 min pre-GA induction	92	152/90 (MAP 107)	100
5 min post-GA induction	90	127/69 (MAP 87)	100
5 min preblock	95	126/68 (MAP 85)	100
5 min postblock	97	119/61 (MAP 81)	100
5 min postincision	90	118/53 (MAP 74)	100
PACU time 00 min	87	127/71 (MAP 89)	100
PACU time 15 min	86	127/71 (MAP 89)	100
PACU time 30 min	95	125/68 (MAP 87)	98
PACU time 45 min	85	134/84 (MAP 100)	98
PACU time 60 min	83	129/83 (MAP 98)	98

*Note:* HR: heart rate beats per minute; BP: blood pressure in mmHg systolic/diastolic; MAP: mean arterial pressure in mmHg; SpO_2_: oxygen saturation in percentage. PACU readings are recorded at 15 min intervals from the time of arrival into PACU.

Abbreviation: PACU, Postoperative Anesthesia Care Unit.

## Data Availability

The data were collected in a retrospective manner from the electronic records and is presented in the manuscript.
